# New Persistent Opioid Use After Surgery

**DOI:** 10.1001/jamanetworkopen.2024.60794

**Published:** 2025-02-20

**Authors:** Razvan Bologheanu, Aylin Bilir, Lorenz Kapral, Felix Gruber, Oliver Kimberger

**Affiliations:** 1 Division of General Anesthesia and Intensive Care Medicine, Department of Anesthesia, Intensive Care Medicine, and Pain Medicine, Medical University of Vienna, Vienna, Austria; 2 Ludwig Boltzmann Institute for Digital Health and Patient Safety, Vienna, Austria; 3 The Umbrella Organization of Austrian Social Security Institutions, Vienna, Austria

## Abstract

**Importance:**

New persistent opioid use after surgery is contributing to the opioid crisis affecting the US, and interventions to limit postoperative opioid prescriptions have been proposed to mitigate the opioid-related health care burden. Limited information is available regarding the incidence of new persistent use after surgery in other countries.

**Objective:**

To determine the incidence of new persistent opioid use and to evaluate factors associated with its risk after surgery in Austria.

**Design, Setting, and Participants:**

This retrospective, population-based cohort study used administrative data from the national Austrian social insurance database for adult patients undergoing surgery between January 1, 2016, and December 31, 2021. Exclusion criteria were incomplete exposure or outcome data, early postoperative death, and ongoing opioid use at the time of surgery. Data were analyzed from September 2023 to August 2024.

**Exposures:**

General, gynecological, urological, orthopedic, and cardiac surgery.

**Main Outcomes and Measures:**

The primary study outcome was the incidence of new persistent opioid use up to 6 months after surgery in the Austrian population; the factors associated with use, including patient factors and surgical procedures, were analyzed. A logistic regression model was used to evaluate the association between the primary outcome and the independent variables after controlling for multicollinearity.

**Results:**

Among 559 096 patients undergoing 642 857 surgical procedures (median [IQR] age, 60 [48-71] years; 318 391 male patients [49.5%]), new persistent opioid use was documented in 10 810 cases (1.7%) overall, and the rates ranged from 0.3% for appendectomy (130 cases per 40 565 procedures) to 0.7% for abdominal surgery (2198 cases per 335 034 procedures) to 6.8% for spinal surgery (3495 cases per 51 348 procedures). The median (IQR) daily opioid dose in oral morphine equivalents was 7.4 (4.1-14.9) mg. Specific procedures, including spinal surgery (odds ratio [OR], 5.36; 95% CI, 5.04-5.69) and arthroplasty (OR, 1.57; 95% CI, 1.48-1.67), and patient characteristics, such as previous opioid use that was discontinued before surgery (OR, 3.06; 95% CI, 2.93-3.19) and the frequency of previously filled opioid prescriptions (OR, 16.49; 95% CI, 13.63-19.95), were associated with new persistent opioid use.

**Conclusions and Relevance:**

In Austria, the incidence of new persistent opioid use after surgery is lower than that in North America, but the factors associated with risk are similar. These findings suggest the need for studies comparing pain management strategies, as well as systemic and organizational factors, that contribute to the development of new persistent opioid use after surgery in North American and European health care systems, where less evidence about new persistent opioid use has been available.

## Key Points

**Question:**

What is the incidence of new persistent opioid use after surgery, and what risk factors are associated with it?

**Findings:**

In this population-based study of 559 096 Austrian adults undergoing 642 857 surgical procedures, the incidence of new persistent postoperative opioid use was 1.7%. Spinal surgery, advanced age, prolonged hospital stay, a history of substance use disorder, and previous opioid use that was discontinued before surgery were associated with new persistent opioid use.

**Meaning:**

These findings are consistent with existing literature but suggest the need for additional studies comparing pain management strategies and systemic and organizational factors that contribute to the development of new persistent opioid use after surgery.

## Teaser

This cohort study examines the incidence of and factors associated with the risk of new persistent opioid use after surgery among adults in Austria.

## Introduction

In the context of the current opioid crisis, new persistent opioid use (NPOU) after surgery has emerged as a major concern owing to its possible progression to long-term use and misuse in patients initially prescribed opioids for postoperative analgesia.^1,2^ Although various definitions have been proposed, NPOU usually involves continued use of opioids for at least 3 to 6 months postoperatively, in contrast with the expected postoperative course where acute postoperative pain subsides as the patients recover from surgery.^3^ NPOU is considered both a postoperative complication and a clinical phenomenon that accompanies perioperative care, and its incidence and underlying factors have been particularly well documented in the setting of North American health care.^2,4,5^ Although certain procedures are more frequently complicated by NPOU (eg, orthopedic surgery), even low-risk but common surgical procedures, such as abdominal wall hernia repair, can greatly contribute to postoperative opioid prescriptions.^2,6,7^

NPOU has been recognized as a major contributor to the opioid epidemic, which is a public health emergency in the US, and the associated increase in health care costs, thus prompting reevaluation of postoperative pain management practices.^4,8,9^ Currently, there are few data available on opioid prescribing practices and postoperative opioid use outside North America and UK, where most of the existing research has been conducted.^10,11,12,13^ However, there is a strong rationale for investigating opioid prescriptions and NPOU in health care systems outside North America and UK with different insurance coverage, access to care, prescribing practices, regulatory and cultural issues, and patient management strategies.^14,15^ Upstream measures aimed at limiting the opioid supply, particularly postoperative opioid prescriptions, are considered key interventions to minimize the risk of long-term opioid use.^16^ Therefore, understanding postoperative NPOU in health care systems where these limitations are already in effect is crucial for improving patient outcomes and preventing opioid-related complications following surgical procedures. Addressing this gap in the literature could help differentiate between systemic and clinical factors, potentially leading to more tailored interventions and policies for preventing postsurgical opioid misuse and may suggest improvements applicable both within and across health care systems. The aims of this nationwide cohort study were to determine the incidence of NPOU after surgery and to evaluate clinical factors associated with the risk of NPOU after surgery in the Austrian health care system.

## Methods

### Study Design

This retrospective cohort study used administrative data collected by the Umbrella Organization of Austrian Social Security Institutions (Vienna, Austria), which is the public health insurance providing health care coverage for 98% of residents of Austria (8.6 to 8.8 million residents between 2016 and 2021). The health insurance database includes prescription fills from the outpatient sector for all drugs reimbursed by the insurance providers, including Anatomical Therapeutic Chemical Classification System and clinical data, such as demographics, hospital stay, discharge dates, along with *International Statistical Classification of Diseases and Related Health Problems, Tenth Revision* system–coded diagnoses. The Ethics Committee of the Medical University Vienna, Austria approved this study on April 18, 2023, and waived the requirement for informed consent because the data were deidentified, in accordance with the Declaration of Helsinki.^17^ The manuscript has been prepared in accordance with the Strengthening the Reporting of Observational Studies in Epidemiology (STROBE) reporting guidelines.

### Study Population and Data Collection

Patients undergoing surgery between January 1, 2016, and December 31, 2021, were identified on the basis of the billing codes for selected procedures. The procedures included in the study were abdominal wall hernia repair, appendectomy, cholecystectomy, colectomy, mastectomy and other breast surgery, prostatectomy, thyroidectomy, laryngeal surgery and other head and neck procedures, hysterectomy, spine surgery, coronary artery bypass graft, and knee and hip arthroplasty. Patients who filled an opioid prescription within 3 months before admission, as well as patient records with incomplete outcome or exposure data, were excluded from the study. Accordingly, patients who underwent surgery in the first 3 months in 2016 or the last 3 months of 2021, patients who underwent surgery between July and September 2021 and filled at least 1 opioid prescription in the first 3 months after surgery, and patients who died within 90 days after surgery were excluded.

Patient characteristics (eg, age, sex, residential area, primary diagnoses, surgical procedures, admission and discharge dates, and perioperative analgesic prescriptions, including the date when opioid prescriptions were filled and the dose) were pseudonymized and collected. Opioid prescriptions were identified according to the Anatomical Therapeutic Chemical codes. All opioid medications were included regardless of the approved indication (ie, tramadol, fentanyl, morphine, buprenorphine, methadone and levomethadone, oxycodone, and dihydrocodeine).

### Primary Outcome and Independent Variables

NPOU after surgery was the primary outcome of this study. We used the definition proposed by Clarke et al,^18^ where NPOU is considered to occur whenever at least 1 opioid prescription is filled within the first 90 days after surgery followed by at least 1 prescription filled within the days 90 to 180 after surgery. Explanatory variables included remote opioid use, defined as previous opioid use that was discontinued before surgery, derived from preoperative opioid prescriptions, both as a binary variable and further categorized by recency, mean prescribed opioid dose (oral morphine equivalents), and frequency of opioid prescriptions. We further included patient demographics, residential area, the surgical procedures, including the type of access (open vs laparoscopic) and the anatomical site, and the following associated conditions: chronic pain, psychiatric comorbidities (grouped as schizophrenia, mood disorders, substance use disorder, and neurotic disorders), diabetes, congestive heart failure, chronic obstructive pulmonary disease, and malignant neoplasms.

### Statistical Analysis

Data were analyzed from September 2023 to August 2024. All analyses were conducted using the open-source data science and statistical packages pandas and statmodels for Python statistical software version 3.10.9 (Python Software Foundation). Patients were grouped according to prior opioid use, and descriptive statistics were calculated for all explanatory variables. The incidence of NPOU was computed, and a logistic regression model was used to evaluate the association between the primary outcome and the independent variables after controlling for multicollinearity. Age, the prescribed opioid dose, the frequency of opioid prescriptions, and the hospital length of stay (LOS) were normalized between 0 and 1. The reciprocal value of the time between surgery and the most recent opioid prescriptions (1 divided by the time interval) was normalized—that is, values closer to 1 represented more recent opioid prescriptions. Model selection was performed using Akaike information criterion.

To account for the different preoperative windows of observation and to evaluate their association with the completeness of clinical data, particularly in respect to remote opioid use and previous surgical procedures, sensitivity analyses were performed, first, by adding the preoperative observation time and the time elapsed since the previous surgery to the regression model and second, by fitting the regression model on the dataset overall and stratified by year. Odds ratios (ORs) and 95% CIs were calculated, and statistical significance was defined as a 95% CI that did not contain the null value of 1.

## Results

**Figure 1.**
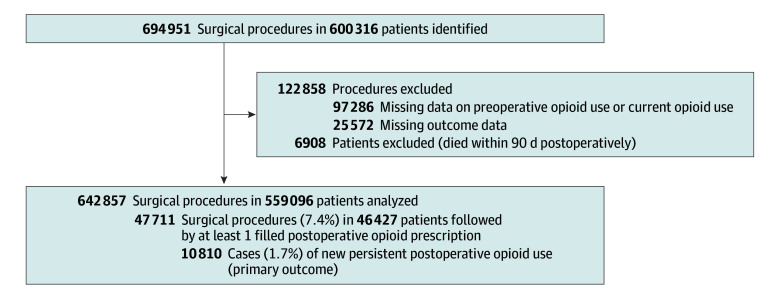
Patient Enrollment Flowchart Adults undergoing abdominal wall hernia repair, appendectomy, cholecystectomy, colectomy, mastectomy and other breast surgery, prostatectomy, thyroidectomy, laryngeal surgery and other head and neck procedures, hysterectomy, spine surgery, coronary artery bypass graft, and knee and hip arthroplasty were screened to be included in this study.

We identified 600 316 patients who underwent 694 951 surgical procedures between 2016 and 2021. After excluding 25 572 procedures with incomplete outcome data and 6908 patients who died within 90 days after surgery, as well as 97 286 procedures for patients taking opioids at the time of surgery or missing data on preoperative opioid use, 642 857 surgical procedures (median [IQR] age, 60 [48-71] years; 318 391 male patients [49.5%]) among 559 096 patients were analyzed. Characteristics of the study participants and procedure type by previous opioid use category are shown in Table 1. In 47 711 cases (7.4%), at least 1 opioid prescription was filled postoperatively. NPOU after surgery was recorded after 10 810 surgical procedures (1.7%) (Figure 1). NPOU incidence ranged from 0.3% for appendectomy (130 cases per 40 565 procedures) to 0.7% for abdominal surgery (2198 cases per 335 034 procedures) to 6.8% (3495 cases per 51 348 procedures) for spinal surgery (Table 2). The median (IQR) daily opioid dose in oral morphine equivalents was 7.4 (4.1-14.9) mg.

**Table 1.** Surgical Procedures and Patient Characteristics

Characteristic	Patients, No. (%)		
	No previous opioid use (n = 576 941)^a^	Remote opioid use (n = 65 916)^b^	Total (N = 642 857)^c^
Age, median (IQR), y	59 (47-70)	65 (54-73)	60 (48-71)
Sex			
Male	290 634 (50.3)	27 757 (42.1)	318 391 (49.5)
Female	286 307 (49.7)	38 159 (57.9)	324 466 (50.5)
Urban area^d^	176 835 (30.6)	20 614 (31.2)	197 449 (30.7)
Substance use disorder	13 051 (2.2)	3260 (4.9)	16 311 (2.5)
Psychiatric comorbidities			
Mood disorders	10 943 (1.9)	4048 (6.1)	14 991 (2.3)
Schizophrenia	1316 (0.2)	190 (0.3)	1506 (0.2)
Neurotic, stress, and somatoform disorders	5487 (0.9)	1658 (2.5)	7145 (1.1)
Other comorbidities			
Chronic pain	71 206 (12.3)	25 339 (38.4)	96 545 (15)
Diabetes	26 405 (4.5)	7076 (10.7)	33 481 (5.2)
Congestive heart failure	8006 (1.4)	2291 (3.4)	10 297 (1.6)
Chronic obstructive pulmonary disease	14 570 (2.5)	4532 (6.8)	19 102 (2.9)
Malignant neoplasm	80 735 (14)	11 464 (17.4)	92 199 (14.3)
Procedure type			
Abdominal surgery	313 099 (54.2)	21 935 (33.2)	335 034 (52.1)
Cardiac surgery	14 256 (2.5)	1279 (1.9)	15 535 (2.4)
Head and neck surgery	55 025 (9.5)	4210 (6.4)	59 235 (9.2)
Spine surgery	39 680 (6.8)	11 668 (17.7)	51 348 (7.9)
Breast surgery	15 906 (2.7)	1144 (1.5)	16 971 (2.6)
Arthroplasty	138 975 (24.1)	25 759 (39)	164 734 (25.6)
Open approach	452 461 (78.4)	57 046 (86.5)	509 507 (79.2)
Hospital length of stay, median (IQR), d	5 (3-9)	7 (4-11)	5 (3-9)

^a^ During the study period, 49 706 patients underwent 2 surgical procedures, and 7776 patients underwent 3 or more surgical procedures.

^b^ During the study period, 6728 patients underwent 2 surgical procedures, and 1255 patients underwent 3 or more surgical procedures.

^c^ During the study period, 60 192 patients underwent 2 surgical procedures, and 10 389 patients underwent 3 or more surgical procedures.

^d^ There were 1570 patients with missing values.

**Table 2.** Incidence of New Persistent Opioid Use After Surgery

Anatomical site and surgical procedure	New cases, No.	Total procedures, No.	Incidence, % (95% CI)
Abdomen			
Abdominal wall hernia repair	579	126 756	0.45 (0.42-0.50)
Appendectomy	130	40 565	0.32 (0.27-0.38)
Cholecystectomy	625	84 452	0.74 (0.68-0.80)
Colectomy	577	26 570	2.17 (2.00-2.35)
Hysterectomy	229	41 475	0.55 (0.48-0.62)
Prostatectomy	58	15 216	0.38 (0.29-0.49)
Breast			
Breast surgery	153	16 971	0.90 (0.77-1.05)
Head and neck			
Laryngeal surgery	35	742	4.71 (3.41-6.48)
Thyroidectomy	146	36 438	0.40 (0.34-0.47)
Other procedures	502	22 055	2.27 (2.08-2.48)
Limb			
Hip arthroplasty	1499	80 468	1.86 (1.77-1.95)
Knee arthroplasty	2503	84 266	2.97 (2.85-3.08)
Spine			
Surgery of the spine	3495	51 348	6.80 (6.59-7.02)
Heart			
Coronary artery bypass graft	279	15 535	1.79 (1.59-2.01)

### Factors Associated With Risk of NPOU

**Figure 2.**
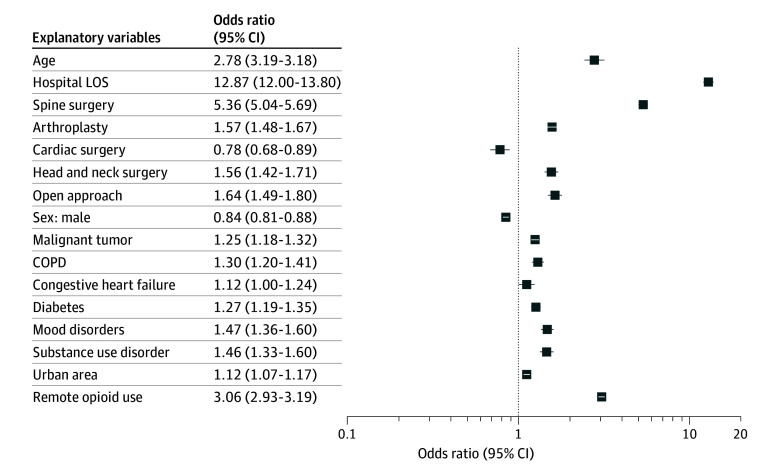
Patient and Surgical Factors Associated With New Persistent Opioid Use Figure shows results of multivariable regression analysis of new persistent opioid use after surgery. COPD indicates chronic obstructive pulmonary disease; and LOS, length of stay.

A total of 642 857 observations and 16 variables were included in the final regression model. NPOU after surgery was independently associated with several patient characteristics and surgical factors. The ORs for all explanatory variables included in the regression model are presented in Figure 2.

Remote opioid use was independently associated with the primary outcome (OR, 3.06; 95% CI, 2.93-3.19), as well as advanced patient age (OR, 2.78; 95% CI, 2.43-3.18) and a history of substance use disorder (OR, 1.46; 95% CI, 1.33-1.60). Surgical factors independently associated with the primary outcome included surgery of the spine (OR, 5.36; 95% CI, 5.04-5.69), the hospital LOS (OR, 12.87; 95% CI, 12.00-13.80), hip and knee arthroplasty (OR, 1.57; 95% CI, 1.48-1.67), and open approach surgery (OR, 1.64; 95% CI, 1.49-1.80). Of note, male patients (OR, 0.84; 95% CI, 0.81-0.88) and patients undergoing cardiac surgery (OR, 0.78; 95% CI 0.68-0.89) had lower odds of developing NPOU after surgery, although the effect sizes were small.

### Remote Opioid Use Patterns and NPOU

Opioid use patterns were significantly associated with NPOU, the primary outcome of our study, whereas the prescribed opioid dose preoperatively did not have a significant association with the incidence of NPOU after surgery (eFigure 1 in Supplement 1). The frequency of preoperative opioid prescriptions was associated with much greater odds of NPOU (OR, 16.49; 95% CI, 13.63-19.95) compared with the time since the most recent preoperative opioid prescription (OR, 3.04; 95% CI 2.82-3.29).

### Sensitivity Analysis

Sensitivity analyses confirmed the results of the initial regression model. Adding the length of the preoperative observation window as explanatory variable did not significantly alter the regression model. Stratified analyses by year yielded consistent results (eFigure 2 in Supplement 1). The incidence of NPOU ranged from 2.1% (1946 cases per 92 119 procedures) in 2016 to 1.1% (929 cases per 83 290 procedures) in 2021. Spinal surgery, arthroplasty, and breast surgery were consistently associated with NPOU across all strata, as well as increased patient age, longer hospital LOS, and history of opioid use and substance use disorders.

## Discussion

In this nationwide cohort study that included all patients covered by the Austrian public social insurance system undergoing various surgical procedures between 2016 and 2021, the incidence of NPOU after surgery was lower than that reported in North American studies.^2^ The overall incidence was 1.7%, but interestingly, the incidence by procedure ranged from 0.3% for appendectomy to 6.8% for surgery of the spine. Unsurprisingly, the proportion of patients who filled an opioid prescription after surgery and the mean daily dose were also lower compared with the existing literature. These results are in line with available data on worldwide opioid prescriptions, which highlight striking differences between the number of prescriptions and the dose prescribed across different countries and regions, and a higher opioid consumption in North America compared with Europe.^15,19^

The dataset included procedures from both ends of the opioid use risk spectrum, but in all categories, the incidence of NPOU was lower than that reported in the US studies. For example, according to Brummett et al,^2^ the overall incidence of NPOU was estimated at 4.1% to 7.1%, depending on the payer types. Other studies^7,20^ have found that after high-risk procedures, such as total hip or knee arthroplasty, as many as 1 in 5 patients develop NPOU. Although recent studies^21,22^ suggest that prescribing patterns and patient factors might impact prolonged opioid use more than the type of surgery and postoperative pain, certain surgical procedures were found to be associated with increased risk of persistent opioid use in our study. Patients undergoing spine surgery, arthroplasty, and head and neck surgery were more likely to continue using opioids after discharge.

Certain comorbid conditions were also significantly associated with postoperative opioid use. In our cohort, chronic pain and psychiatric comorbidities, particularly mood disorders and substance use disorders, were consistently associated with NPOU after surgery. Our study adds to the body of literature documenting the complex interactions among cooccurring substance abuse, chronic pain, and mood disorders, whose underlying mechanisms cannot be determined from these data.^22,23^ In patients with remote opioid use, the odds of NPOU after surgery were significantly higher, compared with opioid-naive patients. Agarwal et al^24^ first described the effect of remote exposure in a secondary analysis of a prospective pain study with a relatively small sample size, but our investigation confirmed their results in a much larger cohort and found a larger effect size (OR, 3.06; 95% CI, 2.93-3.19). Furthermore, we were able to demonstrate that not only is remote opioid use associated with NPOU, but also the previous use patterns are significantly associated with opioid-related outcomes after surgery. The association of the prescriptions’ frequency was greater than the association of their recency, whereas the prescribed dose was not associated with NPOU. These findings, which build on the report by Agarwal et al,^24^ could help stratify patients who have been previously exposed to opioids by the type of previous use to better estimate the risk of adverse opioid-related outcomes.

The main implication of our study is that NPOU is most likely determined by both systemic and clinical factors. Even in the context of a more restrictive opioid prescribing culture, NPOU is affecting opioid-naive patients, who are typically considered less challenging than opioid-tolerant patients with respect to postoperative analgesia. We were able to confirm that patients who are not taking opioids at the time of surgery but had a remote exposure to opioid analgesics have a higher risk of continued opioid use, and more frequent and more recent opioid use are the most significant aspects of remote previous opioid use as a factor associated with the risk of NPOU. Despite an overall low incidence of continued opioid use after surgery, previously described factors underlying NPOU had a similar association with postoperative opioid use in our cohort.^22^ Our study suggests that a comparative evaluation of prescribing patterns and patient outcomes between different health care systems should be considered to further understand this clinical phenomenon affecting the postoperative course of many surgical patients. In turn, this will allow clinicians to better guide postoperative opioid prescribing and implement pain management strategies that do not increase the opioid-related health burden.

### Limitations

We acknowledge several limitations of our study. First, our study only includes administrative data. Including granular clinical data on postoperative pain management strategies, such as regional blocks and opioid-sparing strategies used, and pain scores would greatly improve the validity and interpretability of our results. Still, the size of the cohort and the broad spectrum of surgical procedures included support the generalizability of our findings regarding the factors associated with risk of NPOU. Second, we did not differentiate between inpatient and outpatient surgery and could not include data on in-hospital analgesia. The hospital LOS and in-hospital opioid use might play an important role. In-hospital opioid use is subject to tighter control by health care practitioners. Furthermore, organizational and cultural factors account for large differences in the hospital LOS for similar procedures in different countries.^25,26^ Although it was associated with the primary outcome in our cohort, the hospital LOS might be a surrogate for complicated postoperative course. However, given the magnitude of this association, the role of outpatient surgery and the hospital LOS should be addressed in further research. Third, we included data on filled opioid prescriptions, which likely informs the primary outcome. However, the number of prescriptions that were not filled and the actual opioid use are not known. In the US, where the incidence of NPOU is high, up to 75% of all surgical patients will fill an opioid prescription postoperatively, but excess opioid prescribing and unused analgesics supply are common.^19,27,28^ On a similar note, in the absence of patient-reported opioid use, we could only include filled opioid prescriptions that were reimbursed in the dataset and could not account for postoperative illicit opioid use. Fourth, the preoperative observation period and the duration of the follow-up varied by the year of the surgery. The patients who underwent surgery earlier had a shorter preoperative observation window, but a longer follow-up, and vice versa. Although we excluded all patients with uncertain outcome data (ie, follow-up insufficiently long to determine NPOU), this limitation is relevant for the remote opioid use, where in some cases, only relatively recent opioid use (ie, not more than 6 years before surgery for the longest observation period) could be determined.

## Conclusions

In this Austrian nationwide cohort study of 559 096 patients undergoing 642 857 surgical procedures, the incidence of postoperative NPOU and the average opioid doses prescribed were low. Patients who were exposed to opioids remotely had a higher risk of developing NPOU, as well as patients with mood disorders and substance use disorder. Further studies should investigate the quality of postoperative analgesia, as well as prescription patterns and actual opioid use in the Austrian health care system, along with other system-related factors, such as hospital discharge practices to guide opioid prescribing and analgesic management. This research will help streamline current efforts to reduce the opioid-related burden after surgery.

## Notes

Supplement 1. **eFigure 1.** Multivariable regression analysis of new persistent opioid use after surgery with patterns of previous opioid use as explanatory variables **eFigure 2.** Multivariable regression analysis of new persistent opioid use after surgery: odds ratios by year

Supplement 2.
Data Sharing Statement
