# Supplementary material for: New Persistent Opioid Use After Surgery
Source: JAMA Netw Open. 2025 Feb 20;8(2):e2460794. doi: 10.1001/jamanetworkopen.2024.60794 (PMC11843354; doi:10.1001/jamanetworkopen.2024.60794)
Supplement: Supplement 1. — eFigure 1. Multivariable regression analysis of new persistent opioid use after surgery with patterns of previous opioid use as explanatory variables eFigure 2. Multivariable regression analysis of new persistent opioid use after surgery: odds ratios by year [file jamanetwopen-e2460794-s001.pdf]

# Supplemental Online Content

Bologheanu R, Bilir A, Kapral L, Gruber F, Kimberger O. New persistent opioid use after surgery. *JAMA Netw Open*. 2025;8(2):e2460794. doi:10.1001/jamanetworkopen.2024.60794

**eFigure 1.** Multivariable regression analysis of new persistent opioid use after surgery with patterns of previous opioid use as explanatory variables

**eFigure 2.** Multivariable regression analysis of new persistent opioid use after surgery: odds ratios by year

This supplemental material has been provided by the authors to give readers additional information about their work.

© 2025 Bologheanu R et al. *JAMA Network Open*.

**eFigure 1.** Multivariable regression analysis of new persistent opioid use after surgery with patterns of previous opioid use as explanatory variables

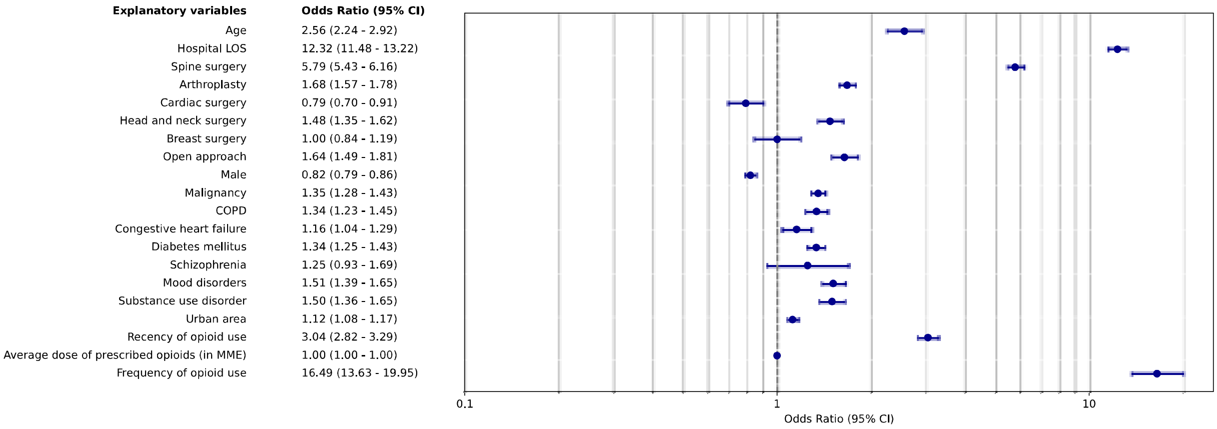


© 2025 Bologheanu R et al. *JAMA Network Open*.

**eFigure 2.** Multivariable regression analysis of new persistent opioid use after surgery: odds ratios by year

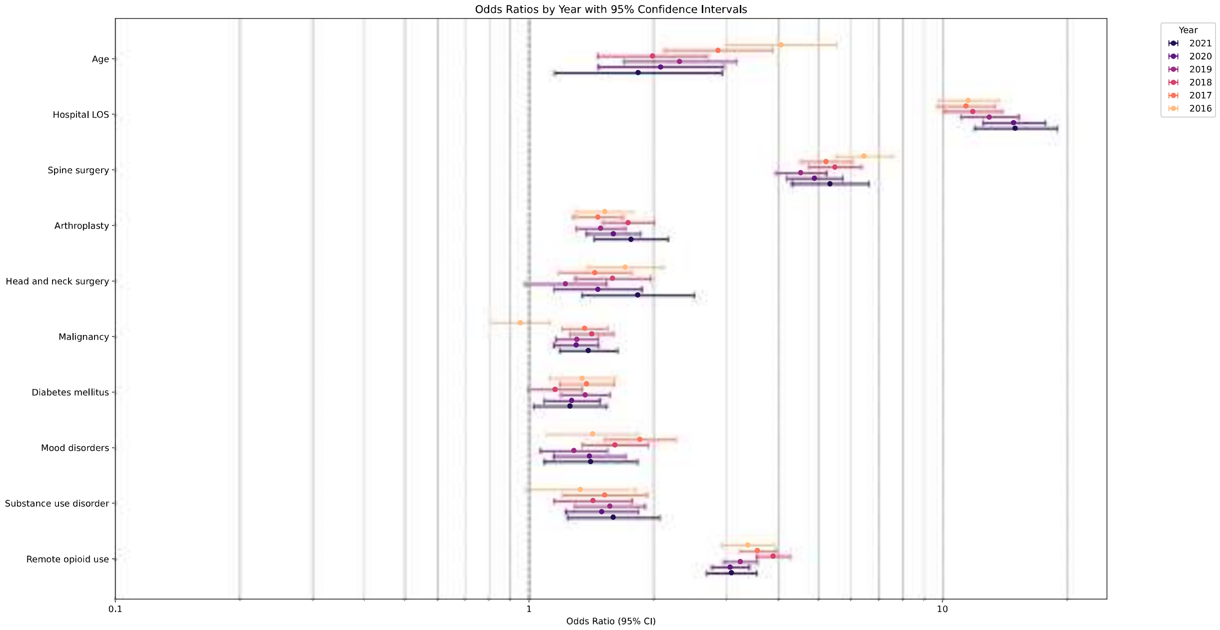


Each color represents a different year, showing the odds ratio and its 95% confidence interval for each explanatory variable across stratified analyses.

© 2025 Bologheanu R et al. *JAMA Network Open*.
