# Supplementary material for: New Persistent Opioid Use After Surgery
Source: JAMA Netw Open. 2025 Feb 20;8(2):e2460794. doi: 10.1001/jamanetworkopen.2024.60794 (PMC11843354; doi:10.1001/jamanetworkopen.2024.60794)
Supplement: Supplement 2. — Data Sharing Statement [file jamanetwopen-e2460794-s002.pdf]

Data Sharing Statement

Bologheanu. New Persistent Opioid Use After Surgery. *JAMA Netw Open*. Published February 20, 2025. doi:10.1001/jamanetworkopen.2024.60794

Data
Data available: No

Additional Information
Explanation for why data not available: The data used for this study is owned by the Umbrella Organization of Austrian Social Security Institutions. Its use for research was made possible and is regulated by a legal agreement between the Medical University of Vienna and the Dachverband, which limits how this data can be used and disseminated. Individual data cannot be made publicly available.
